# Pupal size as a proxy for fat content in laboratory-reared and field-collected *Drosophila* species

**DOI:** 10.1038/s41598-022-15325-0

**Published:** 2022-07-27

**Authors:** Thomas Enriquez, Victoria Lievens, Caroline M. Nieberding, Bertanne Visser

**Affiliations:** 1 Evolution and Ecophysiology Group, Department of Functional and Evolutionary Entomology, Gembloux Agro-Bio Tech, University of Liège, Passage des Déportés 2, 5030 Gembloux, Belgium; 2 Evolutionary Ecology and Genetics Group, Earth and Life Institute, UCLouvain, Croix du Sud 4-5, 1348 Louvain-la-Neuve, Belgium

**Keywords:** Ecology, Ecophysiology, Entomology

## Abstract

In arthropods, larger individuals tend to have more fat reserves, but data for many taxa are still missing. For the vinegar fly *Drosophila melanogaster*, only few studies have provided experimental data linking body size to fat content. This is rather surprising considering the widespread use of *D. melanogaster* as a model system in biology. Here, we hypothesized that fat content in *D. melanogaster* is positively correlated with body size. To test this, we manipulated the developmental environment of *D. melanogaster* by decreasing food availability. We then measured pupal size and quantified fat content of laboratory-reared *D. melanogaster*. We subsequently measured pupal size and fat content of several field-caught *Drosophila* species. Starvation, crowding, and reduced nutrient content led to smaller laboratory-reared pupae that contained less fat. Pupal size was indeed found to be positively correlated with fat content. The same correlation was found for field-caught *Drosophila* pupae belonging to different species. As fat reserves are often strongly linked to fitness in insects, further knowledge on the relationship between body size and fat content can provide important information for studies on insect ecology and physiology.

## Introduction

Body size is a key life history trait in insects^1–4^. Many experimental studies consequently use body size measurements as a proxy for fitness (e.g.^5–8^), because a larger size generally leads to a higher fecundity^9,10^ and longevity^6,9^. Insect body size depends on the environmental conditions experienced during development, such as temperature^11^, nutrition^12,13^ or population density^14^. As insects do not grow as adults, body size is determined entirely during the juvenile stages^15^. Final body size is thus reached after pupation or after the final molt for insects with complete or incomplete metamorphosis, respectively^16–19^. During the development of holometabolous insects, developing larvae need to reach several weight limits for metamorphosis to occur, i.e., the minimal viable weight and the critical weight^20,21^. The minimal viable weight corresponds to the weight at which nutrient reserves, such as fat, are sufficient to survive after metamorphosis, while the critical weight corresponds to a threshold after which metamorphosis can no longer be delayed, even if the larva is starved^20^. In *Drosophila melanogaster,* the minimal viable weight and the critical weight are occurring almost simultaneously during larval development^20^. Starvation before reaching the minimal weight impedes the initiation of metamorphosis, leading to death of the larva, while reduced food intake or starvation after reaching the critical weight leads to smaller adult individuals^20,22^. Reduced nutrition during development thus generally decreases size.

A range of traits are correlated to body size (i.e., allometry), including the relative dimensions of body parts, as well as physiological and behavioral traits^23^. In arthropods, one such key trait is the amount of fat reserves used for energy storage^19,24–30^. Lipids are essential macronutrients for nearly all living organisms, and most animals have the capacity to synthesize and store lipids when resources are abundant^31^. In insects, lipids are stored as lipid droplets in adipocytes of the fat body, mostly as triglycerides, i.e., fat^31,32^. The fat body is an organ present in arthropods that consists of loose tissues mostly located in the abdomen. The fat body is involved in numerous metabolic functions and plays a key role in storage and release of lipids, with a function similar to the liver in vertebrates^31^. In insects, most lipids are accumulated during the larval stage and fat content generally peaks right before pupation^33,34^. Part of the fat reserves are then used for metamorphosis (~ 35% in *D. melanogaster*^34^; up to 50% in the fruit fly *Ceratitis capitata*^35^). Fat reserves available at the onset of the adult stage thus depend on the amount of fat accumulated during the larval stage and the amount consumed during metamorphosis^33,34^. For adult insects, fat reserves can have a large effect on fitness, as high fat reserves can positively affect key life history traits, such as longevity^36^ and fecundity^24,25^. Lipid reserves further play a key role for many other functions, such as stress resistance, survival during overwintering^37^, resistance to drought and starvation^36,38,39^, and increased immunity^40^.

Several studies have investigated the link between fat content and size in *Drosophila melanogaster*. For instance, Bryk et al.^41^ investigated the role of a protein (MAP4K3) on the regulation of body size in *D. melanogaster*, showing that flies where MAP4K3 was knocked down were smaller and had a lower fat content than controls. In another study, Gasser et al.^42^ created lines selected for high mortality and compared body size and fat content with lines selected for low mortality conditions. Only slight differences were observed, but generally the high-mortality selected flies were smaller and leaner than flies from the control line. Chippindale et al.^43^ also compared artificially selected lines, with unselected flies being smaller and containing less fat compared to individuals selected for starvation resistance. Kristensen et al.^44^ selected flies for 17 generation on a high protein diet and observed that these individuals had a greater body size and contained more fat than their counterparts selected on a standard diet. Juarez-Carreño et al.^45^ studied the role of candidate genes in body-fat sensing, and showed that larvae where the gene *Sema1a* (a gene regulating lipid transport and ribosome maturation) was knocked down were bigger and contained more fat than control larvae. These mutants were, however, unable to pupate and initiate metamorphosis. All these studies indeed report that smaller individuals have lower fat reserves (or conversely that bigger individuals have higher fat reserves), but for each of these studies treatments were compared to a control and variation in fat content was not directly correlated to size. Overall, relatively few studies have determined the relationship between body size and fat content in adult *D. melanogaster,* and immature developmental stages are rarely studied. This lack of knowledge is surprising given that *D. melanogaster* is a widely used model species in biology^46–49^, and a promising emerging model for studying lipid metabolism and obesity^50–55^.

To the best of our knowledge, no data yet exists on the relationship between size and fat content of non-mutant *Drosophila* prior to emergence. Here, we aimed to test whether fat reserves and size are positively correlated in *Drosophila* pupae. We further aimed to establish a non-invasive method to estimate pupal fat content (i.e., without destructive sampling) based on size*.* By manipulating developmental conditions, experienced by *D. melanogaster* larvae reared under laboratory conditions, in terms of nutrient content and availability, we produced a gradient of pupal sizes that were subsequently measured for total fat content. To be able to expand our findings to more ecologically relevant conditions, we further collected wild *Drosophila* species and tested for a correlation between pupal size and fat content. Our results show that there is a strong positive correlation between pupal size and fat content, both in laboratory-reared and field-caught *Drosophila.* Estimates of pupal size thus provide a good proxy for pupal fat content in several *Drosophila* species.

## Methods

### Insect maintenance and developmental conditions

Our *Drosophila melanogaster* (Diptera: Drosophilidae) stock originated from a culture that was set up in 1994 from field collections in Sainte-Foy-les-Lyon (France), kindly provided by Patricia Gibert (Claude Bernard University, Lyon, France) in 2016. Larvae were maintained in flasks with continuous access to food medium (60 ml/flask; composition: 20 g agar, 35 g yeast, 50 g sugar, 5 ml nipagin containing 100 g 4-methyl hydroxyl benzoate in 1 l 96% alcohol, and 5 ml propionic acid per liter water). After emergence, adults were maintained in cages (50 × 50 × 50 cm) with continuous access to the same food medium that was replaced every 3 to 4 days. Individuals were kept at a temperature of 23 °C, a relative humidity of 75%, and a photoperiod of L:D 16:8, unless stated otherwise.

To generate pupae that varied in size, we manipulated nutrient content or availability during fly development using three methods: starvation, crowding, and modification of the sugar content in the medium. To do so, flies were allowed to lay eggs during 24 h. Approximately 100 eggs were then collected using a fine paintbrush and distributed in vials (containing 10 ml food medium, a surplus quantity of food to avoid competition between larvae) that differed in nutrient content. To change the nutrient content of the food medium, the standard medium (described above; denoted as 1/1 = 1 part sugar/1 part yeast) was modified to contain either twice more (i.e., 2/1; n = 3 vials) or no sugar (i.e., 0/1; n = 1 vial). As a control, 4 vials of the standard 1/1 medium were also prepared. Nutrient availability was altered by allowing 100 larvae per vial to feed on the standard 1/1 medium for 2 or 3 days. Larvae were then starved by transferring them to a new vial containing a medium without sugar or yeast (starvation after 2d and 3d, respectively; n = 3 vials for both treatments). A third treatment was added where the number of individuals per vial was increased, leading to crowding, and hence a reduction in nutrient availability. To create crowding conditions, 300 eggs were counted under a stereomicroscope and transferred to a vial containing 1 ml of the standard 1/1 medium. This egg density (300 eggs ml^−1^) was chosen, because previous work showed that *D. melanogaster* experiences strong crowding under these conditions^56^. All vials were inspected twice a day for newly formed pupae to ensure that pupae were collected within one day after pupation. Pupae were collected individually, their development time recorded, and then frozen at − 20 °C until further processing. Only rarely vials could not be inspected within 24 h, which was taken into account in the statistical analyses (see below).

### Collection of *Drosophila* pupae from the field

In addition to manipulating the size of laboratory-reared *D. melanogaster*, we aimed to investigate the correlation between size and fat content of wild *Drosophila* individuals. To do so, we collected individuals from the field using banana-bait traps. Each trap consisted of a 0.75 l plastic box, with an opening in the lid. The opening was covered by a fine net mesh of ~ 1 mm. Three traps were prepared, each containing half a banana, as well as a mixture of live baker’s yeast and apple cider vinegar. Each trap was then attached to a tree with the opening facing downward for 1 week in a backyard in Leuven (Belgium). Temperature and humidity were monitored with a thermometer-hygrometer Ibutton (Maxim Integrated) placed inside one of the traps (Supplementary Fig. 1a). For each trap, bananas containing eggs and larvae were placed in a flask containing the standard 1/1 food medium (three flasks in total). In addition to banana-bait traps, we also collected cherries infested by *Drosophila suzukii* from a cherry tree at the same location in Leuven. Cherries were distributed among 6 flasks containing the standard 1/1 food medium. All flasks were then kept in a cage and placed outside our facility (facing North and in continuous shadow). Temperature and humidity inside the cage were also monitored using an Ibutton device (Supplementary Fig. 1b). Pupae were subsequently collected as described above for laboratory-reared flies. Visual species identification based on pupae is very difficult for most drosophilid species, but the pupal shape of *D. suzukii* is easily recognizable^57^. *Drosophila* obtained from cherries were, therefore, identified as *D. suzukii* based on the shape of their respiratory tubes. Pupae obtained from banana-bait traps remained largely unidentified (referenced hereafter as “other species”). To have an estimation of the species present in each trap, adults were collected, killed and stored at − 20 °C, after which each species was identified^58,59^. Adult *D. melanogaster*, *D. simulans*, *D. hydei* and *D. subobscura* were present in the traps.

### Pupal size measurement and neutral lipid quantification

To measure pupal size, a similar procedure as described in Ref.^18^ was followed. In short, each pupa was photographed individually using a camera linked to a stereomicroscope (Leica, SAPO). The total area of the pupal case was then measured using FiJi software (ImageJ v2.1.051;^60)^. Quantification of the neutral lipid fraction (i.e., fat or triglycerides) was done using the protocol described in Ref.^61^. Briefly, pupae were dried in an oven at 60 °C for 3 days, after which dry weight was determined using a microbalance (Mettler Toledo, MT5). Each pupa was then placed into a glass tube with 4 ml of diethyl ether for 24 h. Pupae were then dried again at 60 °C for 3 days and pupal dry weight determined again, giving the neutral lipid-free dry weight. The absolute amount of fat (total neutral lipids in µg/pupae) was then obtained by subtracting the lipid-free dry weight from the lipid-containing dry weight. Ether extraction is an efficient method for fat quantification, as it extracts predominantly neutral lipids^62^, including triglycerides that represent lipids for energy storage^31^.

### Statistical analyses

All analyses were performed with R version 4.0.2^63^. For laboratory-reared pupae, time to pupation, pupal size, and fat content were analyzed using generalized linear mixed-effects models (GLMM) with poisson (pupation time) or gamma (size and lipid content) error distributions. For all GLMMs, the fixed effect diet was analyzed using analysis of deviance with the “Anova” function from the “car” package^64^. Differences between diet groups were then identified using estimated marginal means comparisons (EMMs) using the “emmeans” function^65^. Vial number and pupa collection time were included as random factors (for pupation time, pupal size, and lipid content), as was the extraction run (for fat content). For each GLMM, models were simplified by removing random factors step by step, and compared using an anova with the “model.sel” function from the “MuMIn” package^66^. When models did not differ significantly and the AIC was smaller (delta AIC ≥ 2), the simplified model was kept based on methods of model simplification presented in Ref.^67^. The final model for the time to pupation included vial number as a random factor and the final model for fat content included pupae collection time and extraction run as random factors. No random factor was included in the final model for size. Final models are presented in Table 1.

**Table 1:** Final models used (after model simplification) for statistical analysis in R.

Data analyzed	Final model used
Time to pupation	glmer(Time ~ Diet + (1|Vial), family = poisson())
Pupal size	glm(Size ~ Diet), family = Gamma())
Pupal fat content	glmer(Fat content ~ Diet + (1|Collection_time) + (1|Run_extraction), family = Gamma())
Pupal size/fat content correlation for laboratory-reared pupae	lmer(Fat content ~ Size × Diet + (1|Vial))
Pupal size/fat content correlation for wild pupae	lmer(Fat content ~ Size × Species + (1|Vial) + (1|Collection_time) + (1| Run_extraction))

The correlation between pupal size and fat content was analyzed using linear mixed-effects models (LMM) for laboratory and field-collected pupae separately. For laboratory pupae, the fixed factor diet was used as a co-variable. Vial number, pupae collection time, and extraction run were included as random factors. The LMM was then simplified as described above for GLMMs, and only vial number was kept as a random factor in the final model (Table 1). The LMM was further simplified for interactions between fixed factors. For field pupae, species was used as a co-variable (*D. suzukii* or “other species”). Flask number, pupae collection time and extraction run were included as random factors. The LMM was simplified as described above, but all random effects and interactions were included in the final model (Table 1). For both LMMs, statistical significance of each variable was determined using analysis of deviance. R^2^ were then calculated for both LMMs using the “r.squaredGLMM” function from the “MuMIn” package. For mixed-effects models, R^2^ comes in two types: marginal and conditional. Marginal R^2^ represents the variance explained by the fixed effects of the model, while conditional R^2^ is interpreted as a variance explained by the entire model, including both fixed and random effects. Both the marginal and conditional R^2^ are reported.

## Results

### Developmental conditions affect pupation time, pupal size and lipid content

**Figure 1:**
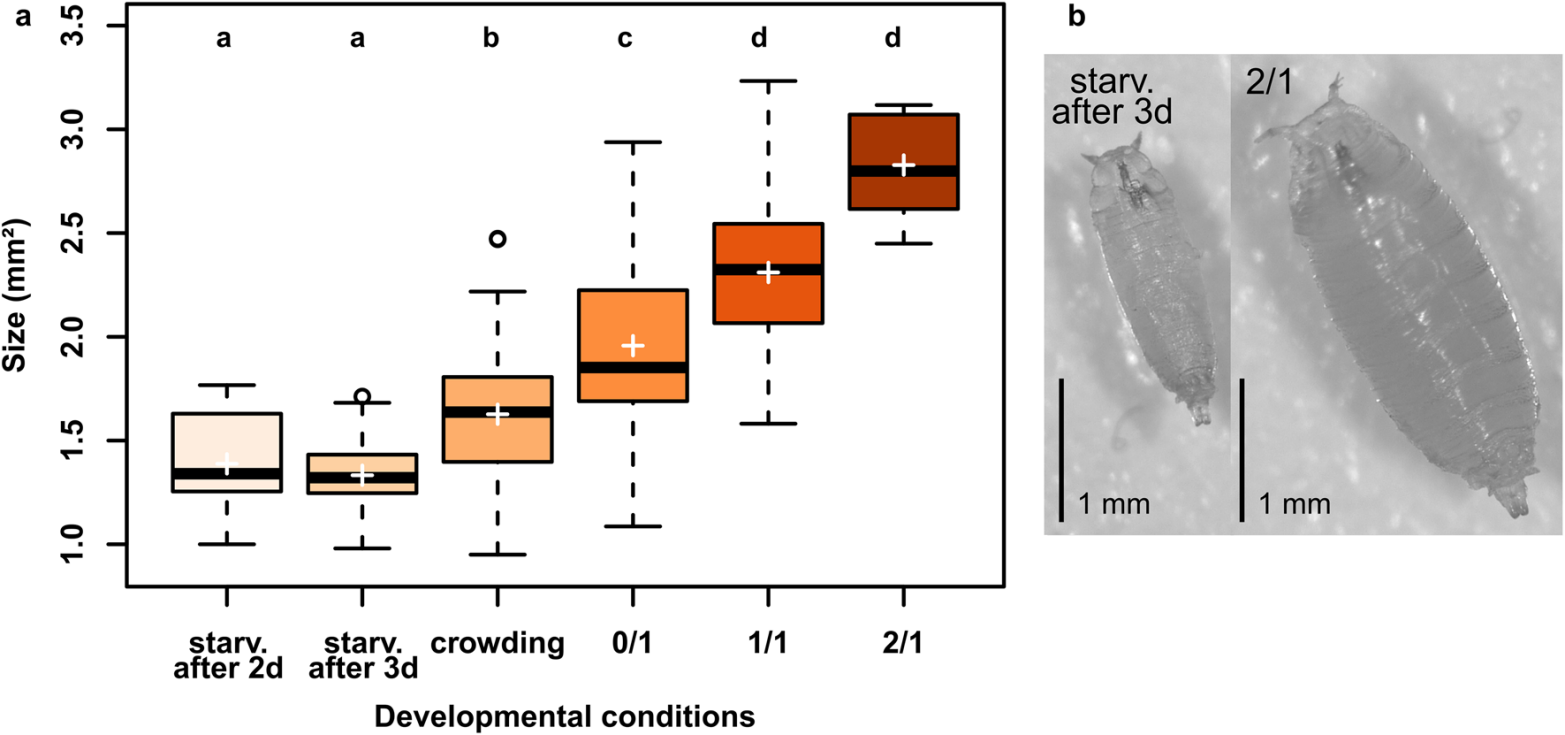
Pupal size (in mm^2^) for laboratory-reared pupae that developed on different diets (**a)**. Picture of a pupa from the high-sugar (2/1) and starvation after 3 days of feeding treatments (**b**). Different letters denote significant differences based on estimated marginal mean comparisons (p value < 0.05). Boxes represent the 25–75 percentile range, horizontal lines display the median and crosses represent the mean. Diets: 1/1: standard medium; 2/1: twice the amount of sugar; 0/1: no sugar; crowding: 300 eggs in 1 ml of standard medium; starv.: starvation after 2 (2d) or 3 days (3d) of feeding on standard medium. Sample sizes are presented in Table 2.

The first aim of this study was to produce pupae of different sizes and fat content. To do so, we manipulated the environmental conditions of developing *D. melanogaster* larvae by decreasing nutrient content or availability of the food medium. All developmental conditions allowed individuals to pupate (see Table 2 for the number of pupae formed per condition, and Supplementary Table 1 for details on the number of pupae formed in each replicate). Diet strongly affected pupal size (Fig. 1; GLMM, χ^2^ = 665.43, df = 5, p value < 0.001). The biggest pupae developed on the high-sugar (2/1; mean ± sd = 2.82 mm^2^ ± 0.49; Fig. 1b) and standard medium (control, 1/1; 2.31 mm^2^ ± 0.32; Fig. 1b), while the smallest pupae were found in the starvation treatments (starvation following 2 or 3 days feeding; 1.38 mm^2^ ± 0.26 and 1.33 mm^2^ ± 0.15, respectively; Fig. 1b). Crowding and the no-sugar medium (0/1) led to pupae of intermediate sizes (1.62 mm^2^ ± 0.29 and 1.95 ± 0.37 mm^2^, respectively; Fig. 1b).

**Table 2:** Number of pupae formed (sample size for size measurement, fat content quantification, and time to pupation), and mean time to pupation (± sd) when development occurred on different diets. Different letters indicate significant differences based on estimated marginal means comparisons (p value < 0.05).

	Starv. after 2d	Starv. after 3d	Crowding	0/1	1/1	2/1
Number of pupae (n)	15	91	96	44	112	17
Mean (± sd) time to pupation (days)	17.73 ± 2.68 (a)	10.57 ± 3.84 (bc)	9.81 ± 1.84 (c)	13.77 ± 1.19 (ab)	9.78 ± 3.30 (cd)	6.64 ± 0.49 (d)

**Figure 2:**
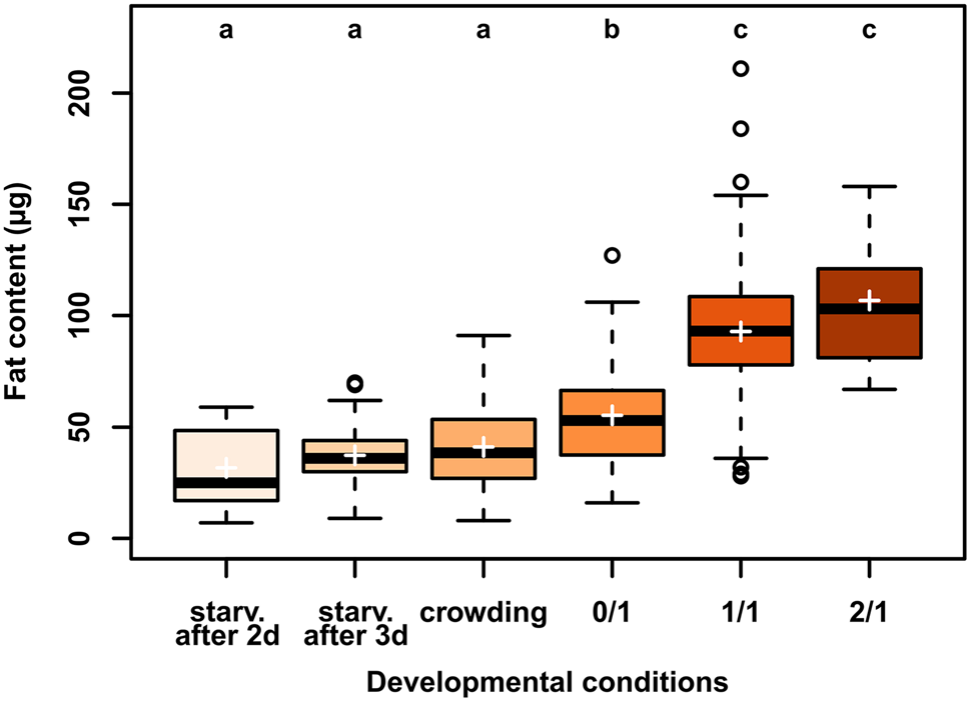
Pupal neutral lipid (i.e. fat) content (in µg) for laboratory-reared pupae that developed on different diets. Different letters denote significant differences based on estimated marginal mean comparisons (p value < 0.05). Boxes represent the 25–75 percentile range, horizontal lines display the median and crosses represent the mean. Diets: 1/1: standard medium; 2/1: twice the amount of sugar; 0/1: no sugar; crowding: 300 eggs in 1 ml of standard medium; starv.: starvation after 2 (2d) or 3 days (3d) of feeding on standard medium. Sample sizes are presented in Table 2.

Diet also had a major effect on pupal fat content (GLMM, χ^2^ = 412.92, df = 5, p.value < 0.001; Fig. 2), with pupae that developed on the nutrient-rich media (2/1 and 1/1; mean ± sd = 106.76 µg ± 26.21 and 92.81 µg ± 30.14, respectively; Fig. 2) showing the highest lipid content, while the leanest pupae (i.e., with the lowest lipid content) were obtained when larvae were starved (starvation after 2d and 3d; 31.73 µg ± 18.58 and 37.36 µg ± 11.31; Fig. 2) and under crowding conditions (41.12 µg ± 18.39; Fig. 2). Pupae developing on the no-sugar medium (0/1) showed an intermediate lipid content (55.34 µg ± 24.49; Fig. 2).

Dietary conditions (nutrient content and/or availability) during development also affected time to pupation, which ranged on average from 6.64 (± 0.49) days for larvae that developed on the sugar-rich (2/1) medium to 17.73 (± 2.68) days for starved larvae following 2 days of feeding (Table 2, GLMM, χ^2^ = 77.196, df = 5, p value < 0.001). Starvation after 2 days of feeding and the no-sugar medium increased development time compared to controls (standard medium, 1/1). Starvation after 3 days of feeding, crowding, and the sugar-rich medium (2/1) did not induce significant changes in development time compared to controls (1/1), but the sugar-rich medium (2/1) shortened development time compared to crowding and starvation after 3 days (Table 2).

### Pupal size and fat content are correlated in laboratory-reared and field-caught *Drosophila* pupae

**Figure 3:**
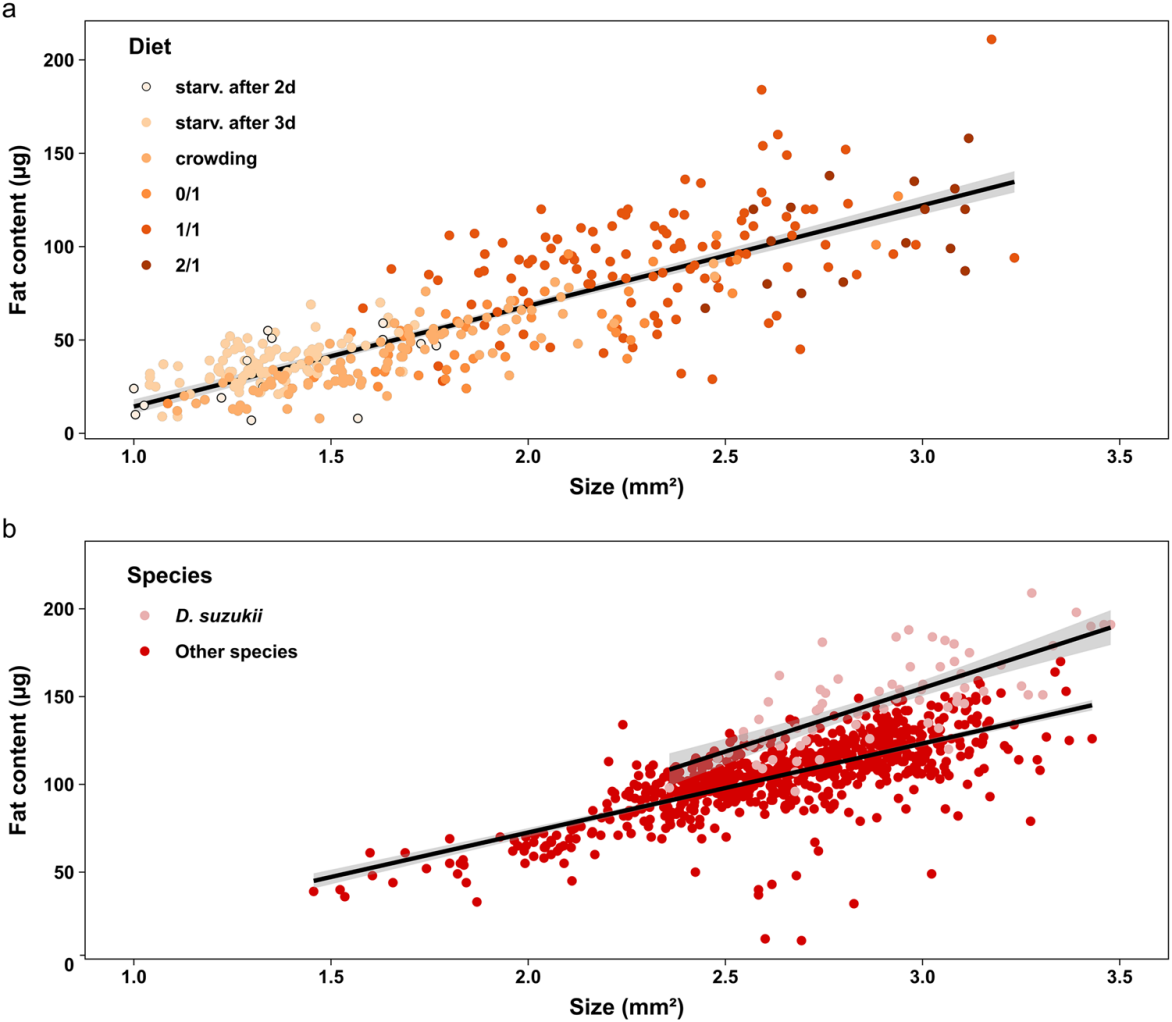
Correlation between pupal size and fat content for laboratory-reared pupae ((**a**), N = 375) and wild pupae ((**b**), N = 810). Lines represent fitted values from the LMMs and the shaded areas represent standard errors. For wild pupae, two regression lines are shown, because there was a significant interaction between pupal size and species (LMM, χ^2^ = 4.13, df = 1, p value < 0.05). Diets: 1/1: standard medium; 2/1: twice the amount of sugar; 0/1: no sugar; crowding: 300 eggs in 1 ml of standard medium; starv.: starvation after 2 (2d) or 3 days (3d) of feeding on standard medium. “Other species” represent pupae collected from banana-bait traps, where several different drosophilids were caught (*D. melanogaster*, *D. simulans*, *D. hydei* and *D. subobscura*).

We used the variation in pupal size and fat content produced by the different environmental conditions of developing *D. melanogaster* larvae to test for a positive correlation between the two traits. Pupal size and fat content were strongly correlated for laboratory-reared individuals, with bigger pupae containing more fat (LMM, χ^2^ = 190.86, df = 1, p value < 0.001, marginal R^2^ = 0.57, conditional R^2^ = 0.85, N = 375, Fig. 3a). Diet did not affect the correlation between pupal size and fat content, meaning that the correlation coefficient was similar when different diets were compared (LMM, χ^2^ = 2.47, df = 5, p value = 0.78).

We further measured pupal size and fat content of several field-caught *Drosophila* species (*D. suzukii* pupae, n = 78; other species, n = 732; see Supplementary Table 1 for the number of pupae formed in each replicate). For field-collected pupae, we also found a strong correlation between size and fat content (LMM, χ^2^ = 688.08, df = 1, p.value < 0.001, marginal R^2^ = 0.47, conditional R^2^ = 0.78, N = 810, Fig. 3b). Of all species measured, *D. suzukii* pupae contained the most fat (LMM, χ^2^ = 27.43, df = 1, p value < 0.001; Fig. 3b). We further found an interaction effect between pupal size and species, as the slope of the regression was higher for *D. suzukii* pupae compared to the other species (LMM, χ^2^ = 4.13, df = 1, p value < 0.05, Fig. 3b).

## Discussion

In this study, we manipulated nutrient content and nutrient availability of developing *D. melanogaster* larvae to produce phenotypes that differ in pupal size and fat content. Starvation, dietary restriction, and crowding generally increased development time, but reduced pupal size and fat content. These conditions are indeed known to decrease body size and fat reserves in adult *D. melanogaster*^20,68–70^, but also in other insects^71–74^. In this paper, the conditions that produced the smallest and leanest individuals were crowding and starvation. Crowding both decreases food availability (nutrients are consumed by conspecifics) and food quality (e.g., overconsumption of accumulated toxic waste produced by conspecifics in the food)^75^. For starvation, previous work showed that starvation of *D. melanogaster* larvae before 3 days of age (i.e., 70 h) can provoke major hormonal dysregulation that impedes reaching the minimal viable weight to initiate metamorphosis, thus leading to death^20,22^. In our study, indeed, only few individuals pupated under the harshest starvation condition (only 48 h of feeding after egg laying). Crowding and starvation are complex multifactorial stressors that can explain why these treatments led to the smallest pupae. The crowding conditions used in this study (300 eggs for 1 ml of food) represent a high density for developing individuals, but *D. melanogaster* larvae can develop in conditions of up to 1000 eggs for 1 ml of food. Survival, however, decreases dramatically under those conditions (with only 1.25% of individuals pupating^56^). It would be interesting to test how even higher densities than the one used in this study would influence size and fat content of *D. melanogaster.*

Our data further showed no significant differences in size, fat content and development time between controls (1/1) and individuals developing on the sugar-rich medium (2/1). This is in contrast to several studies on *D. melanogaster* that showed that sugar-rich diets increased the fat content of adults^76,77^ and larvae^55^, decreased body size^70,76^, and significantly increased time to pupation^52,77^. High sugar diets can further have deleterious effects on fitness, reducing fecundity^76^ or egg to pupa viability^52^. Absence of significant differences between our sugar-rich medium (2/1) and controls (1/1) can result from the concentration of sugar that was used for our sugar-rich medium (10%). Indeed, in the study of Klepsatel et al.^76^, major differences in body size and fecundity were observed for *Drosophila* that developed on a medium containing 25% sugar. Furthermore, when compared with individuals from the no-sugar medium (0/1), 2/1 pupae were bigger, contained more fat and developed faster, showing that variation in sugar quantity had a significant impact on these life history traits. An increase in fat reserves in relation to sugar quantity in the diet can be expected, because excess sugars from the diet are metabolized through glycolysis and the tricarboxylic acid cycle to produce acetyl coenzyme A (acetyl-CoA). Acetyl-CoA is then converted to triglycerides that are stored in lipid droplets within adipocytes in the fat body^31,78^. Overall, our findings, together with previous studies^20,68–70^, suggest that the nutritional conditions experienced by *D. melanogaster* individuals during the larval stage directly affect the size and fat content of pupae and adults that can have major consequences for fitness.

Pupal and adult size are intimately linked with fitness in insects^2,79^. In *Aedes* mosquitoes, for example, an increase in size of 25% leads to an increase in egg production of 100%^25,80^, while in *D. melanogaster* an increase in size of 43% can increase ovariole numbers by 290%^81^. In moths, larger individuals live longer, with a body size (wing length) increase of 25% leading to an increase of 79% percent in lifespan^82^. Similarly, in *D. pseudoobscura* a 24% increase in size can lead to a 32% increase in longevity^9^, while conversely decreasing sugar proportions in the diet decreases fat content of the flies by 26%, in turn decreasing median longevity by 122%^36^. Having large fat reserves can, however, also comes at a cost. *D. melanogaster* was found to show obesity-like phenotypes, with pathologies similar to those associated with obesity in humans, such as heart (dorsal vessel) failure, decreased endurance or metabolic dysregulation^50,55^. Despite these considerations, higher fitness in *Drosophila* is often associated with size and fat content (females with larger body size and higher fat reserves lay more eggs and live longer). This is clearly demonstrated by numerous studies where individuals artificially selected for a larger body size had higher fitness traits, including female fecundity^10,83^, longevity^84^ or male reproductive success^10,84,85^. The positive relationship between body size and fitness is not limited to insects, but has also been found in vertebrates, including reptiles^86–88^ and mammals^89,90^.

The main aim of this study was to use variation in size and fat content of *Drosophila* pupae (as a consequence of modifying nutritional conditions during developmental) to test the hypothesis that fat content is positively correlated with pupal size. We further collected wild pupae to observe if this correlation was also found in natural *Drosophila* populations. We observed that for field-caught *Drosophila*, fat content of 96% of *D. suzukii* pupae and 100% of pupae from other species were within the range of fat contents produced by our treatments (mainly induced by the nutrient-rich media, i.e., 2/1 and 1/1; see Fig. 3). Validating our hypothesis, pupal fat content was indeed positively correlated to pupal size both in laboratory-reared *D. melanogaster* and field-caught *Drosophila* species. Other works however, showed that some conditions can promote an opposite trend. Indeed, Kristensen et al.^44^ showed that flies developing on a protein enriched medium were larger than controls (measured as dry weight), but had fewer lipid reserves (in % of fly body mass). In our study, we increased the proportion of sugar in the diet, but not the proportion of protein. It would be interesting to test how a gradient of protein concentrations can affect the relationship between body size and total fat content in *D. melanogaster.*

In this study, we did not separate male and female pupae. Yet, we observed a strong relationship between fat content and size, with low variability for both laboratory and field pupae, as highlighted by the high conditional R^2^ (0.85 and 0.78, respectively). In arthropods, females tend to have higher fat reserves than males, but females also generally have a larger body size; hence the relationship between size and fat reserves is expected to be similar for males and females^30^. Pupal size, an easy and reliable measurement, is therefore a robust predictor of fat content for both laboratory-reared and field-caught *Drosophila* species. In insects, several body measurements, such as head width^91^, tibia length^5^, wing length^92^, thorax or abdomen length^93^, and pupal size^17^ are used as a proxy for body size, fat content, and fitness. As insect pupae are easy to handle in general, measuring pupal size represents a convenient and non-invasive method to estimate fat content and potentially fitness. By linking pupal size to fat content, our results can be of interest for future eco-evolutionary and physiological studies, because this method allows to estimate an individual’s energetic reserves with the option to use the same individual for further experimentation and life history measurements.

*Drosophila* larvae and pupae are hosts to numerous parasitic wasp (i.e., parasitoid) species^94^. During development, a parasitoid consumes fat from only a single host insect and fat stores are often not replenished during adult life^95,96^. Host resources available for developing parasitoids can thus in turn have major consequences for adult wasp life history traits^97^. Measuring pupal size of *Drosophila,* therefore, offers the possibility to estimate the amount of fat available for developing parasitoids^98^. Our data can also be of interest from an applied perspective, as the spotted wing drosophila (*D. suzukii*) is a major pest of red berries. Several studies are ongoing to develop biological control methods to fight this pest, such as the sterile or incompatible insect techniques^99–101^. The success of these techniques, which require mass release of sterile/incompatible males in infested areas, relies on the quality of released males^102^. In mass rearing facilities, measurements are made at several critical points to check insect quality. Our data shows that pupal size is a reliable estimate of fat content of *Drosophila* flies, including *D. suzukii*, and could, therefore, represent a good quality measurement of mass-produced individuals.

## Supplementary Information

Supplementary Information.

## Data Availability

All data used on this article are available on demand from the corresponding author.
