# Supplementary material for: Pupal size as a proxy for fat content in laboratory-reared and field-collected Drosophila species
Source: Sci Rep. 2022 Jul 27;12:12855. doi: 10.1038/s41598-022-15325-0 (PMC9329298; doi:10.1038/s41598-022-15325-0)
Supplement: Supplementary file 1 — Supplementary Information. [file 41598_2022_15325_MOESM1_ESM.pdf]

Pupal size as a proxy for lipid content in laboratory-reared and field-collected *Drosophila* species

Thomas Enriquez*[1], Victoria Lievens[2], Caroline M. Nieberding[2] & Bertanne Visser[1]

[1] Evolution and Ecophysiology group, Department of Functional and Evolutionary Entomology, Gembloux Agro-Bio Tech, University of Liège, Passage des Déportés 2, 5030 Gembloux, Belgium

[2] Evolutionary Ecology and Genetics group, Earth and Life Institute, UCLouvain, Croix du Sud 4-5, 1348 Louvain-la-Neuve, Belgium

Contact:* corresponding author, thomas.enriquez@uliege.be

Supplementary Information

Supplementary figure 1: Temperature (°C) and relative humidity (RH, in %) inside the traps during the catching session (a) and in the outside cage during wild pupae development (b).

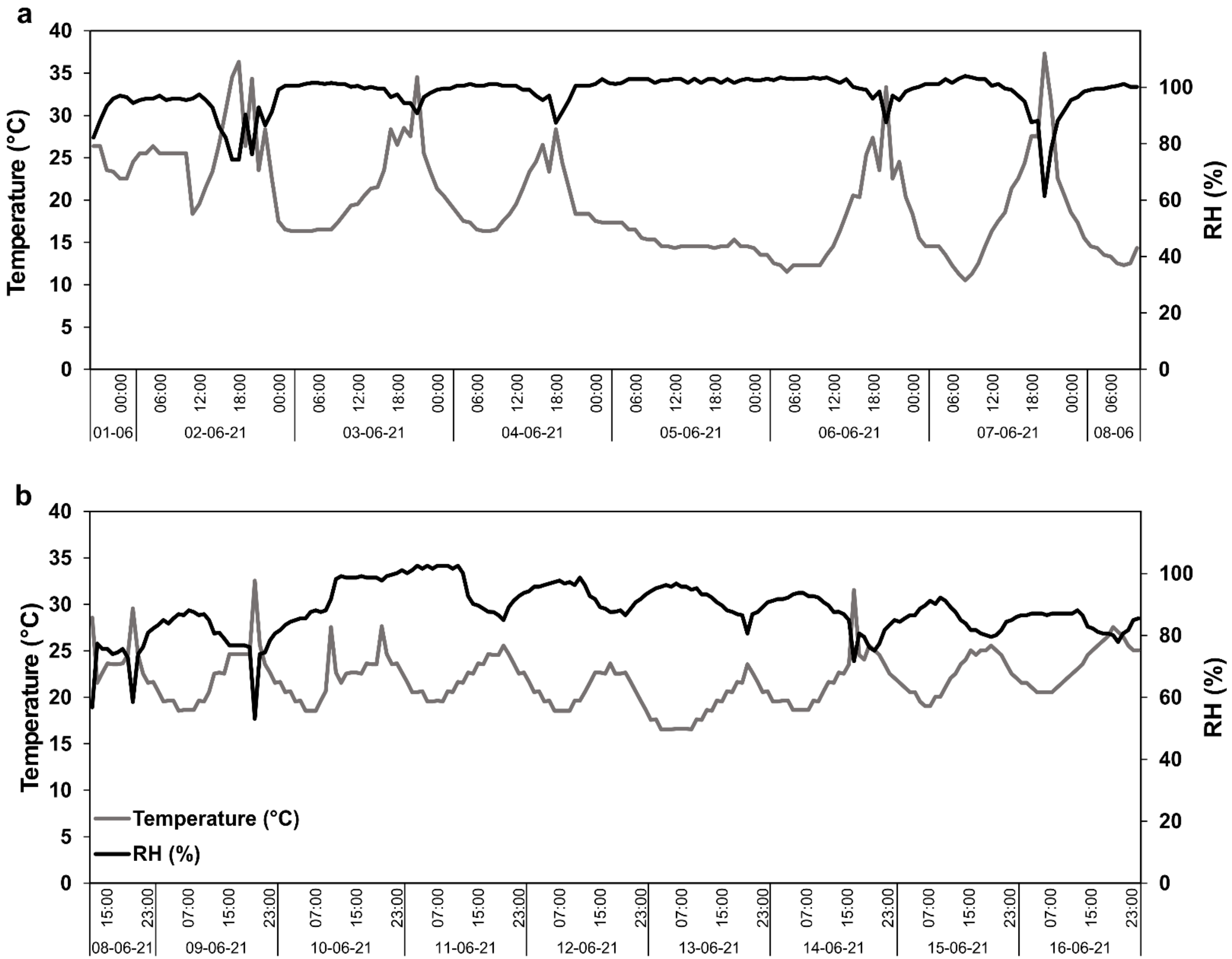

Supplementary Table 1: Number of pupae formed per vial per treatment.

Vial ID	Condition	Number of pupae
1	starv. after 2d	2
2	starv. after 2d	12
3	starv. after 2d	1
1	starv. after 3d	1
2	starv. after 3d	1
3	starv. after 3d	18
4	starv. after 3d	39
5	starv. after 3d	32
1	crowding	96
1	0/1	44
1	1/1	3
2	1/1	8
3	1/1	2
4	1/1	99
1	2/1	7
2	2/1	6
3	2/1	4
1	Banana-bait trap	150
2	Banana-bait trap	200
3	Banana-bait trap	382
1	Infested cherries	5
2	Infested cherries	9
3	Infested cherries	22
4	Infested cherries	3
5	Infested cherries	32
6	Infested cherries	7
